# *Agromyces* *capsici* sp. nov., *Agromyces cucurbitae* sp. nov., and *Agromyces solani* sp. nov.: Three New Species Isolated from Rhizosphere Soils in Yunnan Province, China

**DOI:** 10.3390/microorganisms14081776

**Published:** 2026-08-12

**Authors:** Zhongwen Duan, Shuailiang Shi, Bangli Huang, Mingji Yang, Yongxia Wang, Zefen Yu

**Affiliations:** 1 State Key Laboratory for Conservation and Utilization of Bio-Resources in Yunnan, Yunnan University, Kunming 650504, China; 18348057785@163.com (Z.D.); ssl030727@163.com (S.S.); 18469260684@163.com (B.H.); 15008716509@163.com (M.Y.); 2 Yunnan Key Laboratory of Basic Research and Innovative Application for Green Biological Production, Yunnan University, Kunming 650504, China; 3 School of Life Sciences, Yunnan University, Kunming 650504, China; 4 Yunnan Institute of Microbiology, School of Life Sciences, Yunnan University, Kunming 650504, China

**Keywords:** *Agromyces*, polyphasic taxonomy, genome analysis, rhizosphere soil

## Abstract

Three Gram-stain-positive, aerobic, non-motile, rod-shaped bacterial strains, designated LJC-15^T^, NGA-4^T^ and QZ2-6-4^T^, were isolated from the rhizosphere soils of *Capsicum*, *Cucurbita* and *Solanum melongena*, respectively, in Yunnan Province, China. Phylogenetic and phylogenomic analyses placed the three strains within the genus *Agromyces* but distinguished them from recognized species. All ANI and dDDH values between the novel strains and phylogenetically related type strains were well below the generally accepted species delineation thresholds. The three strains could also be differentiated from selected reference type strains by phenotypic characteristics. Chemotaxonomically, they contained MK-12 as the predominant menaquinone and anteiso-C_15:0_, anteiso-C_17:0_ and iso-C_16:0_ as the major fatty acids, while their whole-cell sugar and polar lipid profiles were consistent with those characteristic of the genus *Agromyces*. On the basis of the combined phylogenetic, genomic, phenotypic and chemotaxonomic evidence, strains LJC-15^T^, NGA-4^T^ and QZ2-6-4^T^ are proposed to represent three novel species of the genus *Agromyces*, for which the names *Agromyces capsici* sp. nov., *Agromyces cucurbitae* sp. nov. and *Agromyces solani* sp. nov. are proposed, respectively. The type strains are LJC-15^T^ (=GDMCC 1.6754^T^ = KCTC 59690^T^), NGA-4^T^ (=GDMCC 1.6755^T^ = KCTC 59691^T^) and QZ2-6-4^T^ (=GDMCC 1.6756^T^ = KCTC 59692^T^), respectively.

## 1. Introduction

The genus *Agromyces*, a member of the family Microbacteriaceae in the phylum Actinomycetota, was established by Gledhill and Casida in 1969 to accommodate a group of Gram-stain-positive soil bacteria with rudimentary branching hyphae [1]. The description of the genus was later emended by Zgurskaya et al. in 1992 to include catalase-positive strains and to refine its chemotaxonomic characteristics [2]. Recent taxonomic studies published in the *International Journal of Systematic and Evolutionary Microbiology* have continued to expand the recognized species diversity of the genus, including the descriptions of *A. chitinivorans*, *A. litoreus*, *A. zhanjiangensis*, *A. endophyticus*, and *A. excoecariae* [3,4,5]. Members of the genus have been reported from diverse habitats, including caves, marine sediments, insect guts, soils, and plant rhizospheres [6,7,8,9,10]. Chemotaxonomically, members of the genus are typically characterized by MK-11 and/or MK-12 as the predominant menaquinones and by anteiso-C_15:0_, anteiso-C_17:0_, and iso-C_16:0_ as the major cellular fatty acids [11,12].

In recent years, the isolation and characterization of novel *Agromyces* species from diverse habitats have broadened our understanding of the ecological diversity and metabolic potential of this genus. Several members of the genus exhibit traits of environmental and biotechnological relevance. For example, *Agromyces* sp. KY5R degrades nylon oligomers via the thermostable enzyme NylC, while *Agromyces* sp. MT-O can mineralize phthalate esters, including di(2-ethylhexyl) phthalate (DEHP) [13,14]. In addition, several tidal flat-derived species, including *A. chitinivorans*, *A. litoreus*, and *A. zhanjiangensis*, have been reported to exhibit salt tolerance and/or chitin-degrading activity, whereas *A. chromiiresistens* is resistant to Cr(VI) and arsenite and harbors genetic determinants that may be involved in aromatic hydrocarbon degradation [3,15]. Other species display diverse ecological interactions. For example, *A. ramosus* exhibits lytic activity against various microorganisms, whereas *A. endophyticus* was isolated from the endosphere of garlic, and genome-based analyses indicated the presence of putative traits associated with phytohormone production and other plant–microbe interactions [4,16]. In addition, *A. mediolanus* has occasionally been associated with opportunistic human infections [17]. Collectively, these findings highlight the ecological versatility and functional diversity of *Agromyces* species and their potential importance in environmental, agricultural, and clinical microbiology.

Recent updates to the proposed minimal standards for the use of genome data in prokaryotic taxonomy recommend integrating high-quality genome sequences, phylogenomic inference, and overall genome relatedness indices with other taxonomic evidence when novel taxa are described [18]. Although 16S rRNA gene sequence analysis remains widely applicable, similarity values may overlap across different taxonomic ranks and do not always accurately reflect species-level genomic relatedness [19]. Genome-based metrics, including average nucleotide identity (ANI) and digital DNA–DNA hybridization (dDDH), are now widely used as key criteria for species delineation, with generally accepted species-level thresholds of 95–96% for ANI and 70% for dDDH [18,20,21]. In addition, phylogenomic analyses generally provide greater resolution than 16S rRNA gene-based phylogenies for resolving relationships among closely related taxa, thereby improving species delineation and taxonomic classification [22].

Despite increasing recognition of the diversity, ecological adaptability, and metabolic potential of *Agromyces*, the culturable species diversity of this genus in agricultural rhizospheres remains insufficiently characterized, particularly in southwestern China. Yunnan Province encompasses substantial topographic, elevational, and environmental heterogeneity, and studies of rhizosphere microbiomes in the region have documented considerable microbial diversity and community differentiation associated with host plants and soil properties [23]. The combination of this environmental heterogeneity and the distinct selective conditions created by different crop rhizospheres may provide diverse microbial niches and favor the occurrence of previously undescribed bacterial lineages. However, the taxonomic diversity of culturable *Agromyces* associated with crop rhizospheres in Yunnan remains poorly investigated. In this study, three putatively novel *Agromyces* strains were isolated from rhizosphere soil samples collected in Yunnan Province, China. Accordingly, this study aimed to determine whether these isolates represent novel species, establish their taxonomic positions within the genus *Agromyces* through an integration of phenotypic, chemotaxonomic, 16S rRNA gene-based phylogenetic, phylogenomic, ANI, and dDDH analyses, and compare their genomic features with those of closely related species to provide a broader genomic context for their taxonomic differentiation.

## 2. Materials and Methods

### 2.1. Sampling and Isolation

The original isolation procedure was aimed at obtaining culturable bacteria from crop rhizospheres. Therefore, LB agar was used as a general-purpose, nutrient-rich medium for bacterial isolation. During subsequent preliminary identification based on 16S rRNA gene sequence analysis, strains LJC-15^T^, NGA-4^T^ and QZ2-6-4^T^ could not be confidently assigned to any recognized *Agromyces* species and were therefore selected for detailed polyphasic taxonomic characterization. Strains LJC-15^T^ and NGA-4^T^ were isolated from the rhizosphere soils of pepper and pumpkin, respectively, collected in Yuanmou County, Chuxiong Yi Autonomous Prefecture, Yunnan Province, China, in January 2025. The sampling coordinates were 25°46′47.2″ N, 101°50′35.1″ E for strain LJC-15^T^ and 25°48′9.1″ N, 101°51′34.5″ E for strain NGA-4^T^. Strain QZ2-6-4^T^ was isolated from eggplant rhizosphere soil collected in Lujiangba (24°56′30.3″ N, 98°53′40.9″ E), Longyang District, Baoshan City, Yunnan Province, China, in December 2024. The physicochemical properties of the rhizosphere soil samples were not determined at the time of sampling. Because the archived soil samples were no longer available, these properties could not be reliably assessed retrospectively.

Bacterial strains were isolated using the serial dilution plating method. A 10 g portion of each soil sample, which had been stored at 4 °C, was added to 90 mL of sterile tap water and shaken on a rotary shaker at 180 rpm for 30 min to obtain a 10^−1^ (*w*/*v*) soil suspension. The suspension was serially diluted 10-fold with sterile tap water to a final dilution of 10^−5^. Aliquots of 100 μL from the 10^−4^ and 10^−5^ dilutions were spread evenly onto LB agar (Tryptone 10 g; Yeast extract 5 g; NaCl 10 g; Agar 20 g; Water 1 L) plates using a sterile spreader. The plates were incubated at 28 °C for 2–3 days and examined daily. Well-isolated colonies were selected and purified by repeated streaking on fresh LB agar plates. Purified strains were preserved at −80 °C as glycerol stocks containing 30% (*v*/*v*) glycerol.

### 2.2. Phenotypic and Chemotaxonomic Characterization

The strains were cultivated on tryptic soy agar (TSA; Tryptone 17 g, Soytone 3 g, NaCl 5 g, Agar 20 g, Water 1 L) plates at 37 °C for 2–3 days. Gram staining was performed using a Gram staining kit (Solarbio, Beijing, China). Motility was assessed by the hanging-drop method using cells harvested during the logarithmic growth phase. Cell morphology and dimensions were examined using transmission electron microscopy (JEM-2100, JEOL Ltd., Akishima, Tokyo, Japan) after negative staining with 2% (*w*/*v*) phosphotungstic acid. Approximately 40–50 cells of each strain were examined at magnifications ranging from 15,000× to 50,000×. Representative micrographs obtained at 30,000×, 30,000× and 20,000× were selected for strains LJC-15^T^, NGA-4^T^ and QZ2-6-4^T^, respectively.

Growth characteristics on various media were evaluated using Reasoner’s 2A agar (R2A; Yeast extract 0.5 g, Peptone 0.5 g, Casamino acids 0.5 g, Glucose 0.5 g, Soluble starch 0.5 g, K_2_HPO_4_ 0.3 g, MgSO_4_·7H_2_O 0.05 g, Sodium pyruvate 0.3 g, Agar 20 g, Water 1 L), nutrient agar (NA; Peptone 10 g, Beef extract 3 g, NaCl 5 g, Agar 20 g, Water 1 L), LB agar, International Streptomyces Project 2 agar (ISP2; Yeast extract 4 g, Malt extract 10 g, Glucose 4 g, Agar 20 g, Water 1 L), and TSA. The temperature range and optimum were assessed on TSA after 3 days at 10–45 °C (5 °C intervals) by visually comparing colony development and overall plate growth. Growth at pH 4.0–12.0 (1.0-unit intervals) and 0–10% (*w*/*v*) NaCl (1% intervals) was assessed in TSB at 37 °C after 3 days using endpoint OD_600_ measurements, and the condition yielding the highest growth was considered optimal [4].

Catalase activity was tested by adding a 5% (*v*/*v*) H_2_O_2_ solution to freshly grown cells and monitoring bubble production within 30 s. Oxidase activity was determined using test strips impregnated with a mixture of equal volumes of 1% (*w*/*v*) N,N-dimethyl-1,4-phenylenediamine monohydrochloride in distilled water and 1% (*w*/*v*) α-naphthol prepared in 95% (*v*/*v*) ethanol. The abilities of the strains to hydrolyze different substrates were tested on TSA plates supplemented with casein (1%, *w*/*v*), starch (1%, *w*/*v*), xanthine (0.5%, *w*/*v*), hypoxanthine (0.5%, *w*/*v*), Tween 20 (1%, *v*/*v*), Tween 80 (1%, *v*/*v*), or chitin (1%, *w*/*v*). Additional phenotypic and enzymatic characteristics were determined using the API 20NE and API ZYM systems (bioMérieux, Marcy-l’Étoile, France) according to the manufacturer’s instructions. All tests were conducted under the incubation conditions specified for the respective assays.

Unless otherwise specified, cells used for chemotaxonomic analyses were grown on TSA at 37 °C for 2 days before harvesting. For cellular fatty acid analysis, cells were harvested under identical culture conditions, and fatty acid methyl esters were extracted, methylated, and analyzed by gas chromatography according to the protocol of Sasser [24]. The analysis was performed using an Agilent 7890 gas chromatograph (Agilent Technologies, Inc., Santa Clara, CA, USA) equipped with the Sherlock Microbial Identification System, version 6.0 (MIDI, Inc., Newark, DE, USA), and the fatty acids were identified using the TSBA6 database. Whole-cell biomass was hydrolyzed according to the method described by Schleifer and Kandler, and the released sugars were analyzed according to Tang et al. [25,26]. For respiratory quinone analysis, cells were grown in TSB at 37 °C for 3 days, harvested, and lyophilized. Respiratory quinones were then extracted and analyzed by high-performance liquid chromatography according to the method of Tamaoka et al. [27]. Polar lipids were extracted according to the method of Minnikin et al. and separated and identified by two-dimensional thin-layer chromatography [28,29,30].

### 2.3. 16S rRNA Gene Phylogeny

Genomic DNA extracts were prepared using the boiling lysis method and used as templates for PCR amplification of the nearly full-length 16S rRNA gene with the universal bacterial primers 27F and 1492R. The resulting amplicons were subjected to Sanger sequencing [31,32]. Forward and reverse sequencing reads were assembled and manually edited using ContigExpress, a component of Vector NTI Suite 6.0 (InforMax, Inc., Bethesda, MD, USA), to generate a consensus 16S rRNA gene sequence for each strain [33]. The obtained sequences were compared with sequences in the EzBioCloud (https://www.ezbiocloud.net) and NCBI GenBank databases (https://www.ncbi.nlm.nih.gov/) to identify the closest phylogenetic neighbors of the novel strains [34,35]. Based on the sequence similarity results, 16S rRNA gene sequences of closely related type strains were retrieved from GenBank, and their names and nomenclatural status were verified using the LPSN [36].

For phylogenetic analysis, the 16S rRNA gene sequences of the novel strains and closely related type strains were aligned using the ClustalW algorithm implemented in MEGA version 12 [37,38]. Phylogenetic trees were reconstructed using the neighbor-joining (NJ) and maximum-likelihood (ML) methods implemented in MEGA 12. The reliability of the inferred tree topologies was evaluated by bootstrap analysis with 1000 replicates [39,40,41]. The Kimura two-parameter model was used to calculate evolutionary distances for the NJ analysis and as the nucleotide substitution model for the ML analysis [42]. To facilitate comparison with the genome-based analyses, the 16S rRNA gene trees included phylogenetically relevant type strains representing validly published species for which publicly available genome assemblies were available.

### 2.4. Phylogenetic and Genomic Analysis

To clarify the phylogenetic positions and genomic features of the three strains, whole-genome shotgun sequencing was performed. Briefly, the three strains were inoculated into TSB and incubated at 37 °C with shaking at 180 rpm for 2–3 days. Cells were harvested by centrifugation, and genomic DNA was extracted using the Wizard^®^ Genomic DNA Purification Kit (Promega Corporation, Madison, WI, USA) according to the manufacturer’s instructions. DNA concentrations were determined using a TBS-380 fluorometer (Turner BioSystems Inc., Sunnyvale, CA, USA), whereas DNA purity was evaluated spectrophotometrically based on the A260/A280 ratio. DNA samples with A260/A280 ratios of 1.8–2.0 and total amounts exceeding 1 μg were used for library construction.

Approximately 1 μg of genomic DNA from each strain was sheared into fragments of approximately 400–500 bp using a Covaris M220 Focused Acoustic Shearer (Covaris, Inc., Woburn, MA, USA). Sequencing libraries were constructed using the NEXTflex™ Rapid DNA-Seq Kit (Bioo Scientific, Austin, TX, USA). Briefly, the fragmented DNA was end-repaired and phosphorylated at the 5′ ends, followed by adenylation of the 3′ ends, adapter ligation, and PCR enrichment of adapter-ligated fragments. The resulting libraries were subjected to paired-end sequencing to generate 2 × 150-bp reads on an Illumina HiSeq X Ten platform (Illumina, Inc., San Diego, CA, USA) by Shanghai Majorbio Bio-pharm Technology Co., Ltd. (Shanghai, China).

The raw sequencing reads were quality-filtered to remove adapter sequences and low-quality reads using the bioinformatic workflow implemented on the Majorbio Cloud Platform. The filtered reads were assembled de novo using SPAdes v4.2.0 [43]. Finally, contigs shorter than 1000 bp were removed using SeqKit v2.13.0, yielding draft genome assemblies for the three strains [44]. Genome completeness, contamination, and strain heterogeneity were evaluated using CheckM v1.2.5 [45].

To further determine the taxonomic positions of the three strains, genome assemblies of phylogenetically relevant type strains were retrieved from the NCBI database. The reference set comprised type strains of validly published species with publicly available genome assemblies that were identified as close relatives in the 16S rRNA gene sequence similarity searches or formed neighboring lineages with the three strains in the 16S rRNA gene-based or phylogenomic trees. Complete pairwise ANI and dDDH values were calculated among all included strains, including comparisons among the three novel strains, between the novel strains and reference type strains, and among the reference type strains themselves. ANI values were determined using the OrthoANIu algorithm implemented in the EzBioCloud service [46]. The dDDH values were estimated using the Genome-to-Genome Distance Calculator (GGDC 3.0; https://ggdc.dsmz.de/ggdc.php, accessed on 10 April 2026) with Formula 2, as recommended for draft genomes [21,36]. The complete pairwise ANI and dDDH matrices were visualized as a combined heatmap using TBtools v2.323, with ANI values presented in the upper triangle, dDDH values in the lower triangle, and self-comparison values of 100% shown along the diagonal [47]. Reference genomes for the phylogenomic and overall genome relatedness analyses were selected from validly published *Agromyces* species for which suitable genome sequences of the corresponding type strains were available. The dataset included taxa that met at least one of the following criteria: (i) they were identified among the closest type-strain matches based on analyses of the 16S rRNA gene sequences using EzBioCloud or BLASTn against GenBank via the NCBI online BLAST service (BLASTN 2.17.0+; National Center for Biotechnology Information, Bethesda, MD, USA); (ii) they were positioned close to the three novel strains in the 16S rRNA gene-based phylogenetic trees; (iii) they were recovered as close relatives in preliminary phylogenomic analyses. Taxa whose names had not been validly published or for which suitable type-strain genome sequences were unavailable were excluded from the genome-based analyses. The phylogenomic tree was reconstructed using a broader set of available genome sequences from validly published *Agromyces* type strains, whereas the ANI and dDDH analyses were performed using a focused subset comprising the three novel strains and 15 phylogenetically relevant type strains. For taxa included in more than one analysis, the same type-strain designations, genome assemblies and accession records were used consistently throughout the manuscript.

In addition to genome-based similarity indices, a phylogenomic tree was reconstructed among the three strains and selected type strains of the genus *Agromyces*. The assembled genomes were annotated using Prokka v1.15.6, and the resulting predicted protein sequences were analyzed using OrthoFinder v2.5.5 to delimit orthologous groups [48,49]. Single-copy orthologues shared by all analyzed genomes were retained and individually aligned using Clustal Omega v1.2.4 [50]. The resulting alignments were then concatenated into a single supermatrix and trimmed using trimAl v1.4 to remove poorly aligned regions [51]. A maximum-likelihood phylogenomic tree was reconstructed using IQ-TREE v1.6.12 under the LG+F+R4 amino acid substitution model, and branch support was evaluated using 1000 ultrafast bootstrap replicates [52].

Putative prophage regions, resistance- and antibiotic-target-associated model matches, and secondary metabolite biosynthetic gene clusters (BGCs) were computationally predicted using PHASTEST v3.0 (https://phastest.ca/, accessed on 5 June 2026), ARTS v2.0 (https://arts.ziemertlab.com/, accessed on 5 June 2026), and antiSMASH v8.0 (https://antismash.secondarymetabolites.org/, accessed on 5 June 2026), respectively [53,54,55].

## 3. Results

### 3.1. Physiology and Chemotaxonomy

**Figure 1:**
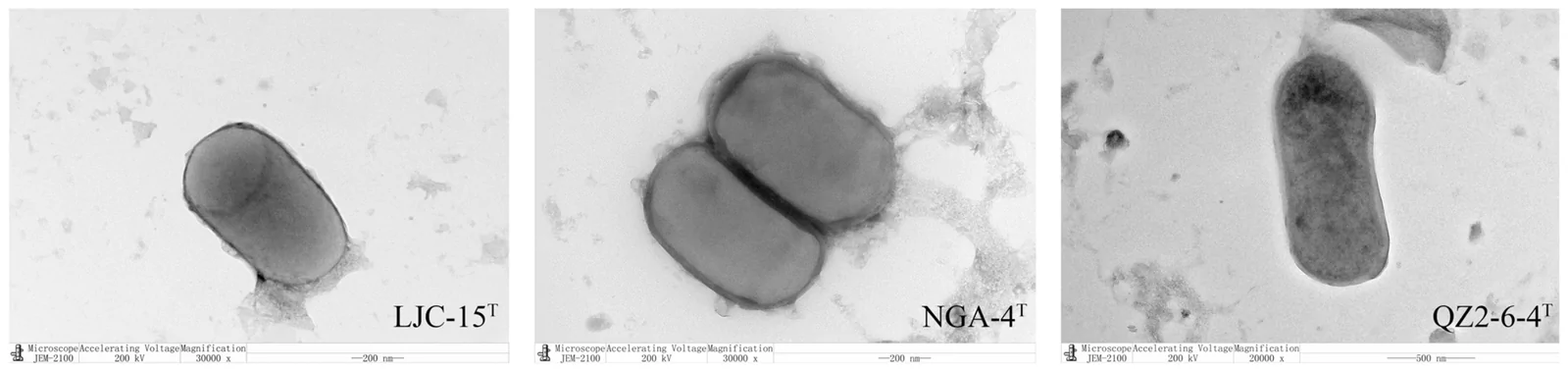
Transmission electron microscopy images of the strains LJC-15^T^, NGA-4^T^, and QZ2-6-4^T^. Scale bars: 200 nm for LJC-15^T^ and NGA-4^T^, and 500 nm for QZ2-6-4^T^.

All three strains were Gram-stain-positive, aerobic and non-motile. Their cells were rod-shaped (Figure 1). They grew on R2A, NA, LB, ISP2 and TSA, with the most abundant growth observed on TSA, where they formed white, circular, smooth and convex colonies after 2 days of incubation. Cell dimensions (width × length) for strains LJC-15^T^, NGA-4^T^ and QZ2-6-4^T^ were 0.3–0.5 × 0.6–1.1 µm, 0.3–0.4 × 0.5–1.0 µm, and 0.4–0.5 × 0.7–1.1 µm, respectively. Strain LJC-15^T^ was able to grow at 10–45 °C (optimum, 35 °C), pH 6.0–9.0 (optimum, pH 7.0), with 0–10% (*w*/*v*) NaCl (optimum, 0%); strain NGA-4^T^ was able to grow at 10–45 °C (optimum, 35 °C), pH 6.0–10.0 (optimum, pH 7.0), with 0–9% (*w*/*v*) NaCl (optimum, 0%); and strain QZ2-6-4^T^ was able to grow at 10–45 °C (optimum, 35 °C), pH 6.0–10.0 (optimum, pH 7.0), with 0–2% (*w*/*v*) NaCl (optimum, 0%). All strains were catalase-positive and oxidase-negative. In terms of hydrolytic activities, all strains were able to hydrolyze hypoxanthine, but not casein, xanthine, Tween 20, Tween 80, or chitin. Starch was hydrolyzed by strains LJC-15^T^ and QZ2-6-4^T^, but not by strain NGA-4^T^. The differential phenotypic characteristics of strains LJC-15^T^, NGA-4^T^ and QZ2-6-4^T^ and selected type strains positioned near them in the 16S rRNA gene phylogeny are summarized in Table 1. The results of API 20NE and API ZYM systems are provided in Table S1. Strain LJC-15^T^ exhibited broader temperature and NaCl tolerance ranges than the selected reference type strains and could also be differentiated by its patterns of enzyme activity, substrate hydrolysis and carbon-source assimilation. Strains NGA-4^T^ and QZ2-6-4^T^ differed from the selected reference type strains in growth ranges and several biochemical characteristics. Furthermore, the two strains could be distinguished from each other by NaCl tolerance, nitrate reduction, starch hydrolysis, α-mannosidase activity, and citrate assimilation, supporting their recognition as phenotypically distinct taxa.

Chemotaxonomically, the major fatty acids of strains LJC-15^T^, NGA-4^T^, and QZ2-6-4^T^ were anteiso-C_17:0_, anteiso-C_15:0_, and iso-C_16:0_ (detailed in Table 2), consistent with the general fatty acid profile of members of the genus *Agromyces*. However, differences in their relative proportions provided additional chemotaxonomic evidence for distinguishing the three strains from closely related species. Whole-cell sugars of all three strains were rhamnose, galactose, mannose, ribose, and glucose, and the predominant menaquinone was MK-12. All three strains contained diphosphatidylglycerol (DPG), phosphatidylglycerol (PG), and two unidentified glycolipids (GLs) as their polar lipids (Figure S1).

### 3.2. Multiphasic Phylogenetic and Phylogenomic Characterization

The 16S rRNA gene sequences of strains LJC-15^T^, NGA-4^T^, and QZ2-6-4^T^ were 1377, 1381, and 1374 bp in length, respectively, and were deposited in the NCBI GenBank database under accession numbers PZ433224, PZ433225, and PZ433226, respectively. The corresponding draft genome sequences were deposited in GenBank under accession numbers JBYRET000000000, JBYRES000000000, and JBYRER000000000, respectively.

Based on 16S rRNA gene sequence comparisons using the EzBioCloud database, the three closest type-strain matches among validly published species were identified for each strain. For strain LJC-15^T^, the closest matches were *Agromyces binzhouensis* OAct353^T^ (98.3%), *A. aurantiacus* YIM 21741^T^ (98.3%), and *A. kandeliae* Q22^T^ (97.9%). For strain NGA-4^T^, the closest matches were *A. mariniharenae* NEAU 184^T^ (98.6%), *A. iriomotensis* IY07-20^T^ (98.4%), and *A. chromiiresistens* H3Y2-19a^T^ (98.3%). For strain QZ2-6-4^T^, the closest matches were *A. chromiiresistens* H3Y2-19a^T^ (98.5%), *A. mediolanus* DSM 20152^T^ (98.5%), and *A. soli* MJ21^T^ (98.4%).

Because the 16S rRNA gene sequence of *A. endophyticus* H17E-10^T^ was not available **in** the EzBioCloud database, additional BLASTn searches against GenBank were performed for strains NGA-4^T^ and QZ2-6-4^T^. Strain NGA-4^T^ showed the highest similarity to *A. endophyticus* H17E-10^T^ (98.9%), followed by *A. mariniharenae* NEAU 184^T^ (98.4%) and *A. iriomotensis* IY07-20^T^ (98.3%). Strain QZ2-6-4^T^ showed the highest similarity to *A. endophyticus* H17E-10^T^ (99.1%), followed by *A. mariniharenae* NEAU 184^T^ (98.5%) and *A. indicus* NIO-1018^T^ (98.3%). Pairwise BLASTn comparisons among the three strains showed sequence similarities of 98.3% between LJC-15^T^ and NGA-4^T^, 98.2% between LJC-15^T^ and QZ2-6-4^T^, and 99.7% between NGA-4^T^ and QZ2-6-4^T^.

**Figure 2:**
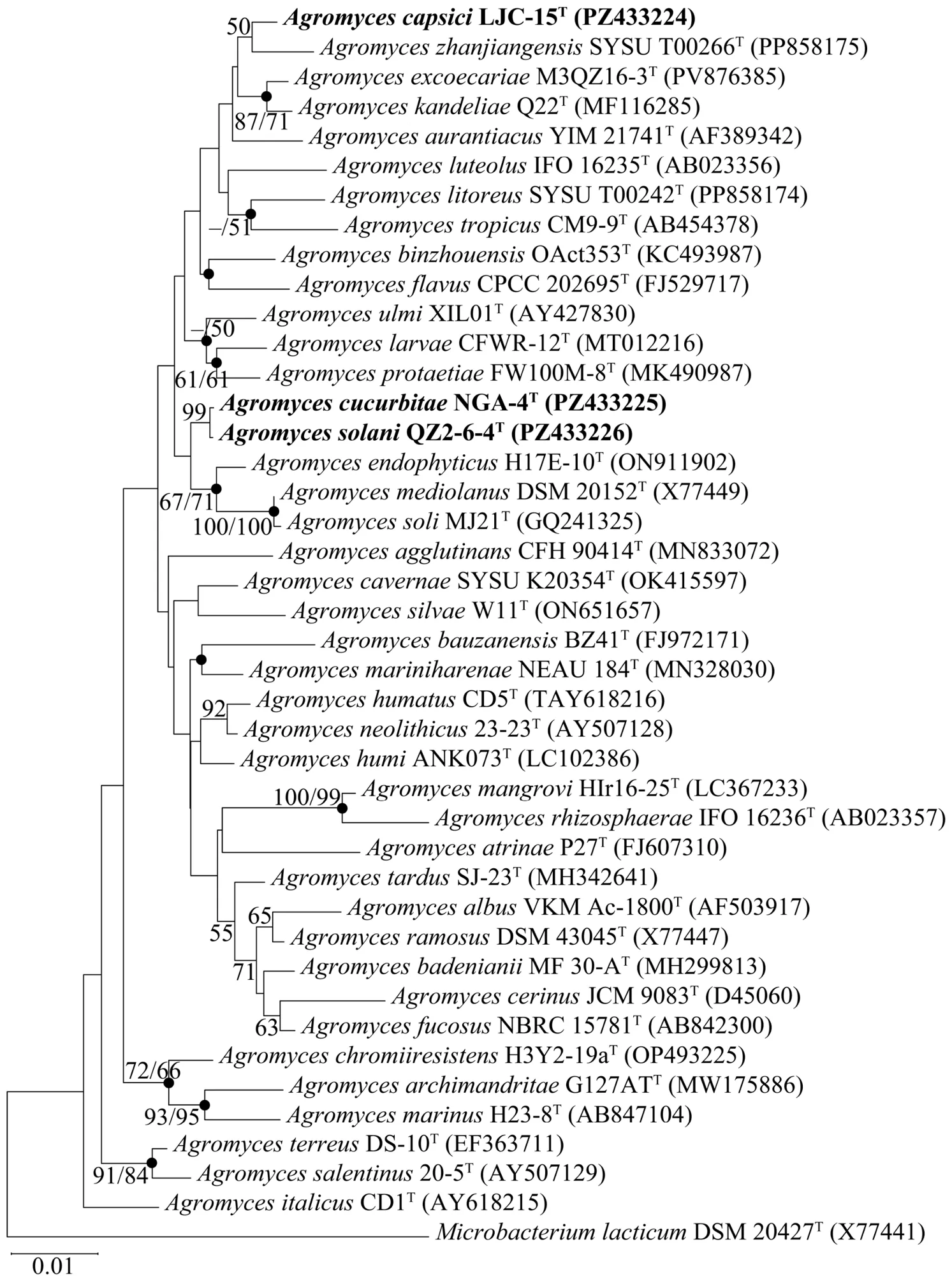
The phylogenetic tree was constructed using the NJ method based on 16S rRNA gene sequences of strains LJC-15^T^, NGA-4^T^, QZ2-6-4^T^ and selected type strains of the genus *Agromyces*. Bootstrap support values (1000 replicates; only values ≥ 50% are shown) are indicated at branch nodes in the format “NJ/ML”, representing support from the NJ and ML analyses, respectively. The three novel strains are shown in bold. *Microbacterium lacticum* DSM 20427^T^ was used as the outgroup. Filled circles indicate nodes recovered in both the NJ and ML trees. A single value denotes support from the NJ method only. A dash indicates that the corresponding node was not recovered by that method. Scale bar, 0.01 substitutions per nucleotide position.

Although 16S rRNA gene sequence similarities between the three strains and their closely related type strains were relatively high, the NJ and ML trees were not fully congruent, and several internal branches received low bootstrap support (Figure 2 and Figure S2). Strain LJC-15^T^ formed a lineage most closely associated with *A. zhanjiangensis*, although this relationship received relatively low bootstrap support. In the NJ tree, strains NGA-4^T^ and QZ2-6-4^T^ formed a closely related pair distinct from the neighboring type strains, whereas this relationship was not recovered with strong support in the ML tree. In addition, the ML topology showed an apparent non-monophyletic arrangement among some of the sampled *Agromyces* lineages. However, because the relevant branches were weakly supported, these placements should be interpreted cautiously and should not be considered conclusive evidence of polyphyly. According to the updated 16S rRNA gene classification boundaries proposed by Hackmann, intraspecies sequence identity can range from 97.2% to 100%, and identity values significantly overlap across different taxonomic ranks [19]. Therefore, a fixed similarity threshold (e.g., 98.65%) should not be used as the sole criterion for species delimitation.

**Figure 3:**
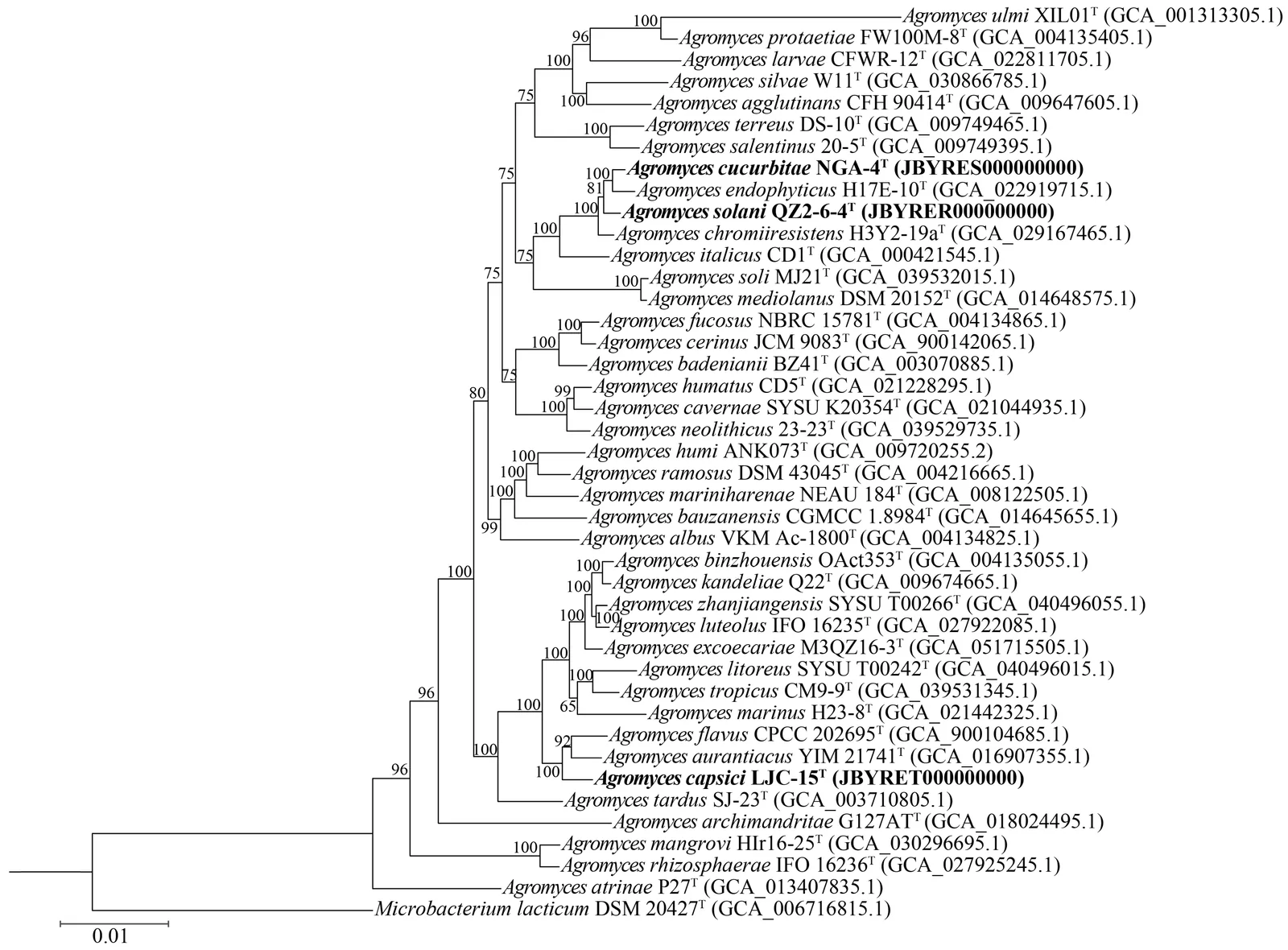
Maximum-likelihood phylogenomic tree reconstructed from the concatenated amino acid sequences of 296 single-copy core orthologous groups shared by strains LJC-15^T^, NGA-4^T^ and QZ2-6-4^T^ and selected type strains of the genus *Agromyces*. The tree was inferred using IQ-TREE v1.6.12 under the LG+F+R4 amino acid substitution model. Numbers at nodes indicate ultrafast bootstrap percentages based on 1000 replicates; only values ≥ 50% are shown. The three novel strains are shown in bold. *Microbacterium lacticum* DSM 20427^T^ was used as the outgroup. Scale bar, 0.01 substitutions per amino acid position.

A maximum-likelihood phylogenomic tree reconstructed under the LG+F+R4 model from the concatenated amino acid sequences of 296 single-copy core orthologous groups shared by the three novel strains and the included type strains provided greater resolution and recovered all three strains as distinct lineages (Figure 3). Strain LJC-15^T^ was recovered as a sister lineage to the clade comprising *A. flavus* CPCC 202695^T^ and *A. aurantiacus* YIM 21741^T^, with 100% bootstrap support for the node uniting LJC-15^T^ with this clade; the *A. flavus*–*A. aurantiacus* pair itself received 92% support. Strain NGA-4^T^ formed a sister relationship with *A. endophyticus* H17E-10^T^ with 81% bootstrap support. Strain QZ2-6-4^T^ was recovered as a sister lineage to this pair with 100% bootstrap support, and this larger clade was sister to *A. chromiiresistens* H3Y2-19a^T^ with 100% support. The topology of the phylogenomic tree was not identical to that of either the NJ or ML tree based on the 16S rRNA gene.

**Figure 4:**
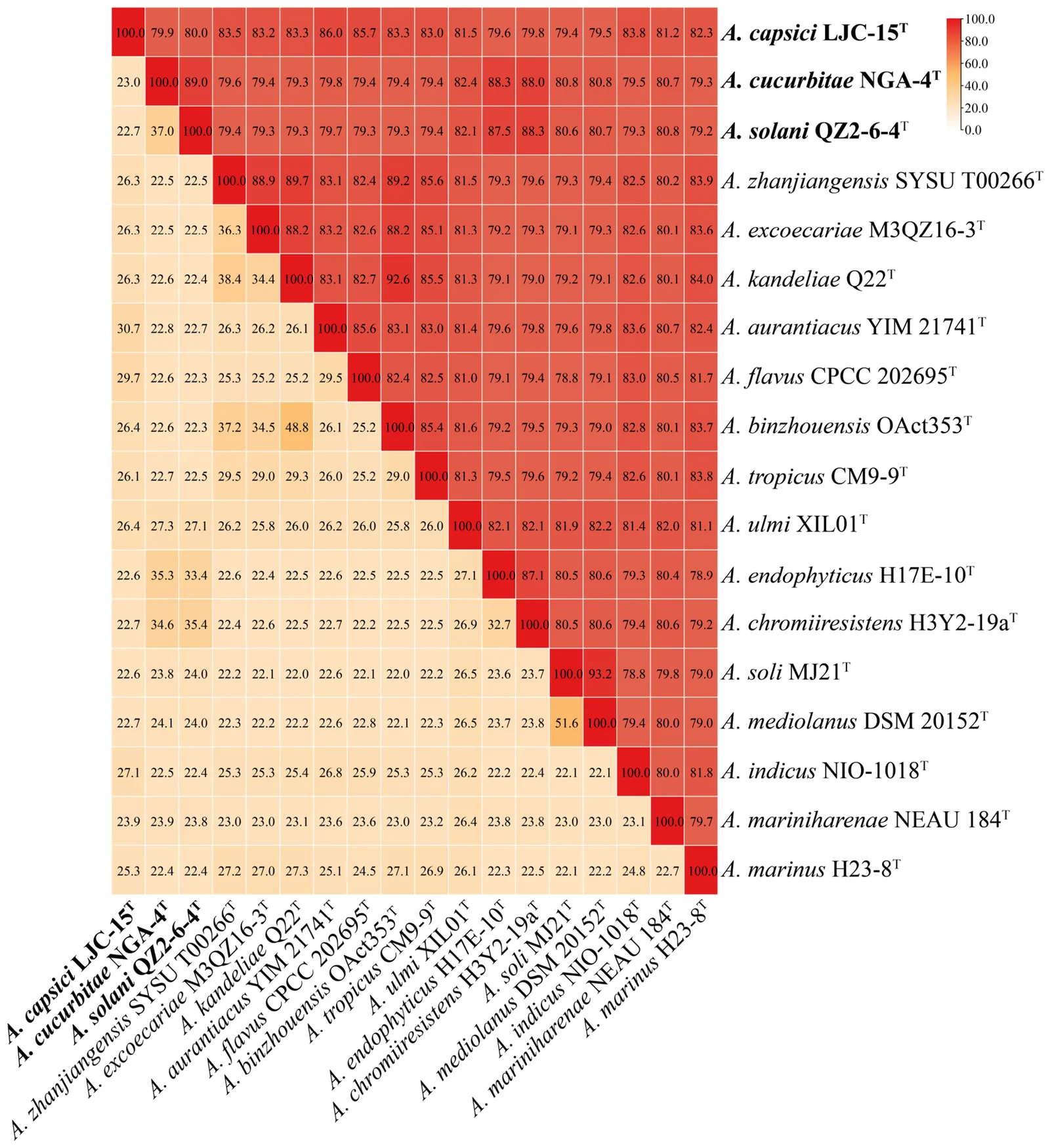
Heatmap of pairwise average nucleotide identity and digital DNA–DNA hybridization values among strains LJC-15^T^, NGA-4^T^ and QZ2-6-4^T^ and phylogenetically relevant type strains of the genus *Agromyces*. ANI values are shown in the upper triangle, whereas dDDH values are shown in the lower triangle. The three novel strains are shown in bold. Self-comparison values are shown as 100% diagonal. Cell colors represent the corresponding percentage values on a continuous scale from 0 to 100. The generally accepted species delineation thresholds are 95–96% for ANI and 70% for dDDH.

Genome-based relatedness was further evaluated using a complete pairwise matrix comprising the three novel strains and 15 phylogenetically relevant type strains (Figure 4). The ANI and dDDH values among strains LJC-15^T^, NGA-4^T^ and QZ2-6-4^T^ ranged from 79.9 to 89.0% and from 22.7 to 37.0%, respectively. Among the reference type strains, LJC-15^T^ showed its highest ANI and dDDH values with *A. aurantiacus* YIM 21741^T^ (86.0% and 30.7%, respectively), NGA-4^T^ with *A. endophyticus* H17E-10^T^ (88.3% and 35.3%), and QZ2-6-4^T^ with *A. chromiiresistens* H3Y2-19a^T^ (88.3% and 35.4%). These genome-relatedness patterns were broadly consistent with the local topology of the phylogenomic tree. In particular, NGA-4^T^ was recovered as the sister lineage of *A. endophyticus*, whereas *A. chromiiresistens*, which showed the highest ANI and dDDH values with QZ2-6-4^T^, was recovered as the sister lineage to the clade comprising QZ2-6-4^T^, NGA-4^T^ and *A. endophyticus*. However, the taxa showing the highest genome-relatedness values were not invariably identical to those showing the highest 16S rRNA gene sequence similarities. All ANI and dDDH values involving the three novel strains were well below the generally accepted species delineation thresholds of 95–96% and 70%, respectively [20,21]. Together with the phylogenetic, phenotypic and chemotaxonomic evidence, these results support the recognition of strains LJC-15^T^, NGA-4^T^ and QZ2-6-4^T^ as representing three novel species of the genus *Agromyces*.

In addition to supporting the distinctness of the three novel strains, the pairwise matrix also revealed several patterns of elevated genome relatedness among the selected reference type strains (Figure 4). For example, *A. zhanjiangensis*, *A. excoecariae* and *A. kandeliae* showed relatively high pairwise ANI values of 88.2–89.7% and dDDH values of 34.4–38.4%, whereas *A. kandeliae* and *A. binzhouensis* showed values of 92.6% ANI and 48.8% dDDH. Likewise, *A. endophyticus* and *A. chromiiresistens* showed 87.1% ANI and 32.7% dDDH, and *A. soli* and *A. mediolanus* showed the highest values among the selected reference taxa, namely 93.2% ANI and 51.6% dDDH. These patterns were broadly consistent with the local topology recovered in the phylogenomic tree (Figure 3).

### 3.3. Genomic Features and Functional Analysis

The genome sizes of the three novel strains ranged from 4.0 to 4.2 Mb, with G+C content of 71.3–71.8%. CheckM analysis showed that the draft genomes of strains LJC-15^T^, NGA-4^T^, and QZ2-6-4^T^ had estimated completeness values of 99.5%, 99.5%, and 98.9%, respectively, and contamination values of 1.0%, 0.5%, and 0.5%, respectively. No strain heterogeneity was detected in any of the three assemblies, indicating that the draft genomes had high completeness and low contamination. The genomic features of the three novel strains and selected reference type strains are summarized in Table 3.

Beyond these general features, genome mining identified several predicted features related to prophages, resistance- and antibiotic-target-associated model matches, and secondary metabolite BGCs, providing targets for future functional investigation.

PHASTEST analysis predicted an intact prophage region only in strain NGA-4^T^ (score, 140; Table S2). The 35.2 kb region, located at positions 552,670–587,917, contained 51 predicted proteins and had a G+C content of 69.0%. The region contained genes predicted to encode proteins associated with prophage integration and excision, DNA replication, genome packaging, virion structure and host-cell lysis, including an integrase, excisionase, DNA primase/polymerase, terminase, portal protein, major capsid protein, tape measure protein, major tail protein, and lysin A (Table S3). A predicted HTH DNA-binding protein (PP_00551), potentially involved in transcriptional regulation, was also identified [57]. The co-occurrence of predicted integrase, excisionase, and regulatory proteins is consistent with a prophage-related genetic module; however, their presence alone does not demonstrate prophage excision, reintegration or the “active lysogeny” mechanism proposed by Feiner et al. [58]. Among the selected phage-associated proteins listed in Table S3, three showed their top BLAST matches to Arthrobacter phage Eileen.

ARTS screening identified resistance- and antibiotic target-associated model matches in all three genomes (Table S4). ARTS model RF0007, associated with ABC efflux transporters, yielded three matches in LJC-15^T^ and NGA-4^T^ and four matches in QZ2-6-4^T^. Although ABC transporters can contribute to the efflux of structurally diverse compounds, their detection alone does not establish multidrug resistance [59]. Models corresponding to DNA gyrase subunit B and DNA topoisomerase IV were also present in all three strains; these conserved proteins are fluoroquinolone targets, but their presence is not evidence of resistance without resistance-associated substitutions or phenotypic susceptibility data [60]. Matches corresponding to chloramphenicol acetyltransferase and VanS were detected only in NGA-4^T^ and QZ2-6-4^T^. These model matches correspond to proteins associated with chloramphenicol inactivation and regulation of vancomycin-resistance systems, respectively, but antimicrobial susceptibility testing is required before resistance phenotypes can be assigned [61,62].

antiSMASH predicted seven, five, and six biosynthetic gene clusters in LJC-15^T^, NGA-4^T^, and QZ2-6-4^T^, respectively (Table S5). In LJC-15^T^, Region 1.2 showed a high-confidence match to an ε-poly-L-lysine biosynthetic gene cluster, whereas its T3PKS and terpene/β-lactone regions showed low-confidence similarities to bisenarsan and carotenoid biosynthetic clusters, respectively. ε-Poly-L-lysine is a cationic antimicrobial polymer with potential applied value, carotenoids are known for antioxidant activity, and bisenarsan has been proposed to act as a prototoxin that releases (2-hydroxyethyl)arsonic acid after hydrolysis [63,64,65]. NGA-4^T^ and QZ2-6-4^T^ shared several predicted BGC types, including hydrogen-cyanide, NRPS-like, terpene, T3PKS, and hybrid β-lactone/terpene-precursor clusters, although the genomic locations and detailed organization of these clusters differed between the two strains. Most predicted clusters showed low or no similarity to characterized BGCs; therefore, their possible products and functions could not be confidently inferred from current database matches and require chemical and genetic validation.

## 4. Discussion

The 16S rRNA gene analyses confirmed the placement of strains LJC-15^T^, NGA-4^T^ and QZ2-6-4^T^ within the genus *Agromyces*, but did not consistently resolve their species-level relationships. The incongruence between the NJ and ML topologies and the low support of several internal branches are consistent with the limited resolving power of this highly conserved single-gene marker among closely related taxa [19]. A comparable pattern was reported for *A. endophyticus*, whose position in the 16S rRNA gene tree differed from that inferred from phylogenomic and overall genome relatedness analyses [4]. In the present study, strains NGA-4^T^ and QZ2-6-4^T^ showed 98.9% and 99.1% 16S rRNA gene sequence similarity to *A. endophyticus*, respectively, whereas the corresponding genome-relatedness values remained far below the accepted species boundaries. In the phylogenomic tree, NGA-4^T^ formed a sister relationship with *A. endophyticus* with 81% bootstrap support, whereas QZ2-6-4^T^ was recovered as sister to this pair with 100% support. Both strains remained distinct lineages, and all of their ANI and dDDH values were well below the species-level thresholds. Therefore, the phylogenomic tree based on 296 single-copy core orthologous groups and the complete ANI/dDDH matrix provide the principal evidence for species-level delineation, while phenotypic and chemotaxonomic differences provide complementary diagnostic support [18,20,21].

The recovery of the three strains from pepper, pumpkin and eggplant rhizospheres extends the cultured taxonomic diversity of *Agromyces* associated with crop-root environments. In comparison, *A. aurantiacus* was isolated from primeval forest soil, whereas *A. soli* was recovered from farm soil [9,12]. *A. chromiiresistens* was isolated from polluted soil and exhibited experimentally determined resistance to Cr(VI) and arsenite, while *A. endophyticus* was isolated from the garlic endosphere and was reported to possess predicted plant-associated traits [4,15]. Comparative genomic analysis of *A. endophyticus* and related taxa, including *A. soli* and *A. chromiiresistens*, also identified transport and metabolism as prominent functional categories [4]. This provides general context for the transport-associated models detected in the three new strains, but does not demonstrate that the specific ARTS matches identified here have equivalent ecological or resistance functions.

The prophage predicted only in NGA-4^T^ may represent a strain-specific acquisition or retention event, or differential loss from related lineages; however, these alternatives cannot be distinguished from the present genome sequences alone. Prophages can influence bacterial genome plasticity and regulation [58]. However, because the present study did not include a genus-wide comparative analysis of prophages, it is not possible to determine whether the predicted region in strain NGA-4^T^ is common or exceptional within the genus *Agromyces*. Likewise, the ARTS matches should be interpreted as genome-mining signals rather than demonstrated resistance determinants, because ABC transporters perform diverse functions in nutrient uptake, metabolite export and stress responses [59]. The predicted BGCs indicate biosynthetic potential rather than confirmed metabolite production. In particular, the putative ε-poly-L-lysine BGC in LJC-15^T^ is a relevant target for future investigation because ε-poly-L-lysine is a cationic antimicrobial polymer with potential applied value [63]. Nevertheless, prophage induction, antimicrobial susceptibility assays, metabolite profiling, gene-expression analysis and controlled plant-inoculation experiments would be required before assigning ecological, agricultural or biotechnological functions to these predicted features.

## 5. Conclusions

The integrated phenotypic, chemotaxonomic, phylogenetic and genome-based evidence supports the recognition of strains LJC-15^T^, NGA-4^T^ and QZ2-6-4^T^ as three novel species of the genus *Agromyces*. Their recovery from different crop rhizospheres expands the knowntaxonomic and genomic diversity of crop-rhizosphere-associated members of the genus. The strain-specific predicted prophage and BGC profiles, particularly the putative ε-poly-L-lysine cluster in LJC-15^T^, provide targets for future functional studies; however, metabolite production, plant-beneficial effects, resistance phenotypes and prophage activity remain to be experimentally evaluated.

### 5.1. Description of Agromyces capsici *sp. nov.*

*Agromyces capsici* sp. nov. (cap′si.ci. N.L. gen. n. *capsici*, of *Capsicum*, referring to the capsicum plant from the rhizosphere soil of which the type strain was isolated).

Cells are Gram-stain-positive, aerobic, non-motile, and rod-shaped, measuring 0.3–0.5 × 0.6–1.1 µm. Colonies on TSA are white, circular, smooth and convex after 2 days of cultivation. Growth occurs at 10–45 °C (optimum, 35 °C), pH 6.0–9.0 (optimum, pH 7.0), with 0–10% (*w*/*v*) NaCl (optimum, 0%). Cells are catalase-positive and oxidase-negative. Starch and hypoxanthine are hydrolyzed, whereas casein, xanthine, Tween 20, Tween 80 and chitin are not hydrolyzed. In the API 20NE system, nitrate reduction, indole production, glucose fermentation, arginine dihydrolase activity, and urease activity were negative, whereas aesculin hydrolysis, gelatin hydrolysis, and β-galactosidase (PNPG) activity were positive. Assimilation tests showed positive reactions for D-glucose, L-arabinose, D-mannose, N-acetyl-D-glucosamine, D-maltose, potassium gluconate, citrate, and phenylacetic acid, while D-mannitol, capric acid, adipic acid, and malic acid were not assimilated. In the API ZYM system, esterase (C4), esterase lipase (C8), leucine arylamidase, valine arylamidase, cystine arylamidase, naphthol-AS-BI-phosphohydrolase, β-galactosidase, α-glucosidase, and β-glucosidase activities were positive. Acid phosphatase and α-mannosidase activities were weakly positive. In contrast, alkaline phosphatase, lipase (C14), trypsin, α-chymotrypsin, α-galactosidase, β-glucuronidase, N-acetyl-β-glucosaminidase, and α-fucosidase activities were negative. The major fatty acids include anteiso-C_17:0_, anteiso-C_15:0_, and iso-C_16:0_. Whole-cell sugars are rhamnose, galactose, ribose, mannose, and glucose, and the predominant menaquinone is MK-12. Polar lipids are DPG, PG, and two unidentified glycolipids (GLs). The G+C content of genomic DNA is 71.8 mol%.

The type strain LJC-15^T^ (= GDMCC 1.6754^T^ = KCTC 59690^T^) was isolated from pepper rhizosphere soil in Yuanmou County, Chuxiong Yi Autonomous Prefecture, Yunnan Province, China. The GenBank accession numbers for the 16S rRNA gene sequence and draft genome sequence of strain LJC-15^T^ are PZ433224 and JBYRET000000000, respectively.

### 5.2. Description of Agromyces cucurbitae *sp. nov.*

*Agromyces cucurbitae* sp. nov. (cu.cur′bi.tae. N.L. gen. n. *cucurbitae*, of *Cucurbita*, referring to the *Cucurbita* plant from the rhizosphere soil of which the type strain was isolated).

Cells are Gram-stain-positive, aerobic, non-motile, and rod-shaped, measuring 0.3–0.4 × 0.5–1.0 µm. Colonies on TSA are white, circular, smooth and convex after 2 days of cultivation. Growth occurs at 10–45 °C (optimum, 35 °C), pH 6.0–10.0 (optimum, pH 7.0), with 0–9% (*w*/*v*) NaCl (optimum, 0%). Cells are catalase-positive and oxidase-negative. Hypoxanthine is hydrolyzed, whereas casein, starch, xanthine, Tween 20, Tween 80 and chitin are not hydrolyzed. In the API 20NE system, nitrate reduction, aesculin hydrolysis, gelatin hydrolysis, and β-galactosidase (PNPG) activity were positive, whereas indole production, glucose fermentation, arginine dihydrolase activity, and urease activity were negative. Assimilation tests showed positive reactions for D-glucose, L-arabinose, D-mannose, N-acetyl-D-glucosamine, D-maltose, and potassium gluconate, while D-mannitol, capric acid, adipic acid, malic acid, citrate, and phenylacetic acid were not assimilated. In the API ZYM system, esterase (C4), esterase lipase (C8), leucine arylamidase, valine arylamidase, cystine arylamidase, naphthol-AS-BI-phosphohydrolase, α-galactosidase, β-galactosidase, α-glucosidase, and β-glucosidase activities were positive. Alkaline phosphatase and acid phosphatase activities were weakly positive. In contrast, lipase (C14), trypsin, α-chymotrypsin, β-glucuronidase, N-acetyl-β-glucosaminidase, α-mannosidase, and α-fucosidase activities were negative. The major fatty acids include anteiso-C_17:0_, anteiso-C_15:0_, and iso-C_16:0_. Whole-cell sugars are rhamnose, ribose, galactose, mannose, and glucose, and the predominant menaquinone is MK-12. Polar lipids are DPG, PG, and two unidentified glycolipids (GLs). The G+C content of genomic DNA is 71.4 mol%.

The type strain NGA-4^T^ (= GDMCC 1.6755^T^ = KCTC 59691^T^) was isolated from pumpkin rhizosphere soil in Yuanmou County, Chuxiong Yi Autonomous Prefecture, Yunnan Province, China. The GenBank accession numbers for the 16S rRNA gene sequence and draft genome sequence of strain NGA-4^T^ are PZ433225 and JBYRES000000000, respectively.

### 5.3. Description of Agromyces solani *sp. nov.*

*Agromyces solani* sp. nov. (so.la′ni. N.L. gen. n. *solani*, of *Solanum*, referring to the plant *Solanum melongena*, from whose rhizosphere soil the type strain was isolated). Cells are Gram-stain-positive, aerobic, non-motile, and rod-shaped, measuring 0.4–0.5 × 0.7–1.1 µm. Colonies on TSA are white, circular, smooth and convex after 2 days of cultivation. Growth occurs at 10–45 °C (optimum, 35 °C), pH 6.0–10.0 (optimum, pH 7.0), with 0–2% (*w*/*v*) NaCl (optimum, 0%). Cells are catalase-positive and oxidase-negative. Starch and hypoxanthine are hydrolyzed, whereas casein, xanthine, Tween 20, Tween 80 and chitin are not hydrolyzed. In the API 20NE system, aesculin hydrolysis, gelatin hydrolysis, and β-galactosidase (PNPG) activity were positive, whereas nitrate reduction, indole production, glucose fermentation, arginine dihydrolase activity, and urease activity were negative. Assimilation tests showed positive reactions for D-glucose, L-arabinose, D-mannose, N-acetyl-D-glucosamine, D-maltose, potassium gluconate, citrate, and phenylacetic acid, while D-mannitol, capric acid, adipic acid, and malic acid were not assimilated. In the API ZYM system, esterase (C4), esterase lipase (C8), leucine arylamidase, valine arylamidase, cystine arylamidase, naphthol-AS-BI-phosphohydrolase, β-galactosidase, α-glucosidase, and β-glucosidase activities were positive. Alkaline phosphatase, acid phosphatase, α-galactosidase, and α-mannosidase activities were weakly positive. In contrast, lipase (C14), trypsin, α-chymotrypsin, β-glucuronidase, N-acetyl-β-glucosaminidase, and α-fucosidase activities were negative. The major fatty acids include anteiso-C_17:0_, anteiso-C_15:0_, and iso-C_16:0_. Whole-cell sugars are rhamnose, ribose, mannose, galactose, and glucose, and the predominant menaquinone is MK-12. Polar lipids are DPG, PG, and two unidentified glycolipids (GLs). The G+C content of genomic DNA is 71.3 mol%.

The type strain QZ2-6-4^T^ (= GDMCC 1.6756^T^ = KCTC 59692^T^) was isolated from eggplant rhizosphere soil in Lujiangba, Longyang District, Baoshan City, Yunnan Province, China. The GenBank accession numbers for the 16S rRNA gene sequence and draft genome sequence of strain QZ2-6-4^T^ are PZ433226 and JBYRER000000000, respectively.

## Figures and Tables

**Table 1:** Differential phenotypic characteristics of strains LJC-15^T^, NGA-4^T^, and QZ2-6-4^T^ and selected reference type strains of the genus *Agromyces*. Only characteristics with taxonomic discriminatory value are shown. Strains: 1, LJC-15^T^; 2, *A. zhanjiangensis* SYSU T00266^T^ [3]; 3, *A. excoecariae* M3QZ16-3^T^ [5]; 4, *A. kandeliae* Q22^T^ [56]; 5, *A. aurantiacus* YIM 21741^T^ [12]; 6, NGA-4^T^; 7, QZ2-6-4^T^; 8, *A. endophyticus* H17E-10^T^ [4]; 9, *A. mediolanus* DSM 20152^T^ [11]; 10, *A. soli* MJ21^T^ [9]. +, positive; −, negative; W, weakly positive; ND, not determined.

Characteristics	1	2	3	4	5	6	7	8	9	10
Temperature range (°C)	10–45	25–37	20–42	10–40	15–40	10–45	10–45	10–40	10–35	10–30
Optimum temperature (°C)	35	37	30	30	28	35	35	30	28	ND
pH range	6–9	6–8	6–11	7–10	6–8	6–10	6–10	7–10	6–9	6–9
NaCl range (%, *w*/*v*)	0–10	0–8	0–6	0–5	0–8	0–9	0–2	0–6	0–3	0–3
**Enzyme activity**										
nitrate reduction	−	−	−	−	+	+	−	+	+	−
alkaline phosphatase	−	+	+	−	−	W	W	−	+	−
lipase (C14)	−	+	W	ND	ND	−	−	−	−	−
trypsin	−	+	−	ND	ND	−	−	−	+	+
α-galactosidase	−	+	+	−	ND	+	W	−	−	−
α-glucosidase	+	−	+	−	−	+	+	+	−	+
α-mannosidase	W	−	−	ND	−	−	W	+	−	−
**Hydrolysis of**										
Starch	+	−	+	+	+	−	+	+	−	−
**Assimilation of:**										
L-arabinose	+	ND	+	+	W	+	+	+	−	−
D-mannitol	−	+	+	+	+	−	−	−	−	−
potassium gluconate	+	ND	+	ND	ND	+	+	+	−	−
Citrate	+	ND	ND	ND	+	−	+	ND	−	−

**Table 2:** Cellular fatty acid compositions (%) of strains LJC-15^T^, NGA-4^T^, and QZ2-6-4^T^ and selected reference type strains. Strains: 1, LJC-15^T^; 2, *A. zhanjiangensis* SYSU T00266^T^ [3]; 3, *A. excoecariae* M3QZ16-3^T^ [5]; 4, *A. kandeliae* Q22^T^ [56]; 5, *A. aurantiacus* YIM 21741^T^ [12]; 6, NGA-4^T^; 7, QZ2-6-4^T^; 8, *A. endophyticus* H17E-10^T^ [4]; 9, *A. mediolanus* DSM 20152^T^ [11]; 10, *A. soli* MJ21^T^ [9]. The three major fatty acids are highlighted in bold. −, not detected; TR, trace amount (<1%).

Fatty Acid	1	2	3	4	5	6	7	8	9	10
iso-C_14:0_	TR	−	1.8	2.3	TR	−	−	3.1	−	1.1
anteiso-C_14:0_	−	−	−	4.7	−	−	−	−	−	−
iso-C_15:0_	6.6	4.7	**17.2**	5.6	8.5	10.8	6.9	4.8	2.0	7.4
anteiso-C_15:0_	**31.0**	**33.5**	**37.8**	**35.3**	**37.3**	**23.8**	**27.1**	**37.5**	**33.0**	**46.9**
iso-C_16:0_	**13.0**	**34.2**	16.0	**32.2**	**20.1**	**11.1**	**11.7**	**31.9**	**20.0**	**18.1**
C_16:0_	2.2	1.0	4.7	1.1	4.2	1.7	3.0	2.3	2.0	1.1
iso-C_17:0_	4.8	TR	3.3	1.9	2.0	8.1	6.0	1.4	−	1.7
anteiso-C_17:0_	**40.9**	**17.0**	**18.5**	**19.8**	**23.6**	**43.6**	**43.8**	**17.9**	**40.0**	**23.7**
C_18:1_*ω*9c	1.1	−	−	−	−	TR	1.5	−	−	−

**Table 3:** Genomic features of the three novel strains and selected reference type strains (Genome assemblies of the reference strains were retrieved from NCBI GenBank, whereas genomic features of strains LJC-15^T^, NGA-4^T^ and QZ2-6-4^T^ were determined in the present study). Strains: 1, LJC-15^T^; 2, *A. zhanjiangensis* SYSU T00266^T^; 3, *A. excoecariae* M3QZ16-3^T^; 4, *A. kandeliae* Q22^T^; 5, *A. aurantiacus* YIM 21741^T^; 6, NGA-4^T^; 7, QZ2-6-4^T^; 8, *A. endophyticus* H17E-10^T^; 9, *A. mediolanus* DSM 20152^T^; 10, *A. soli* MJ21^T^.

Strains	Number of Contigs	N50 (bp)	Genome Size (bp)	GC (%)	CDS	tRNA	rRNA
1	3	2,973,778	4,041,876	71.8	3687	49	5
2	22	369,498	3,901,187	71.8	3618	53	5
3	20	291,607	3,830,412	71.7	3509	51	5
4	22	397,016	3,783,276	71.9	3503	52	2
5	1	3,884,057	3,884,057	72.4	3603	53	6
6	9	964,502	4,216,208	71.4	3785	52	5
7	9	1,029,379	4,083,006	71.3	3702	47	4
8	1	4,250,163	4,250,163	70.9	3790	52	6
9	11	2,064,382	3,861,601	72.4	3572	56	2
10	1	3,810,275	3,810,275	72.2	3554	56	6

## Data Availability

The 16S rRNA gene sequences of strains LJC-15^T^, NGA-4^T^ and QZ2-6-4^T^ have been deposited in GenBank under accession numbers PZ433224, PZ433225 and PZ433226, respectively. Their draft genome sequences have been deposited under accession numbers JBYRET000000000, JBYRES000000000 and JBYRER000000000, respectively. The remaining data supporting the findings of this study are available within the article and its Supplementary Materials.

## Supplementary Materials

The following supporting information can be downloaded at: https://www.mdpi.com/article/10.3390/microorganisms14081776/s1. Figure S1: Polar lipid profiles of strains LJC-15^T^, NGA-4^T^ and QZ2-6-4^T^. Figure S2: Maximum-likelihood (ML) phylogeny inferred from 16S rRNA gene sequences of the three novel strains (LJC-15^T^, NGA-4^T^, and QZ2-6-4^T^) and selected type strains of the genus *Agromyces*. *Microbacterium lacticum* DSM 20427^T^ was used as the outgroup. Bootstrap percentages (1000 replicates) are shown at nodes for values ≥ 50%. The scale bar indicates 0.01 substitutions per nucleotide position. Table S1: Biochemical and enzymatic characteristics of strains LJC-15^T^, NGA-4^T^ and QZ2-6-4^T^ determined using the API 20NE and API ZYM systems. +, positive; −, negative; W, weakly positive. Table S2: A putative prophage region predicted in the genome of strain NGA-4^T^ using PHASTEST. Table S3: Selected phage-associated genes predicted within the putative intact prophage region of strain NGA-4^T^. Table S4: Resistance- and antibiotic target-associated model matches identified in the genomes of strains LJC-15^T^, NGA-4^T^ and QZ2-6-4^T^ using ARTS. Values indicate the numbers of model matches identified in each genome. Table S5: Secondary metabolite biosynthetic gene clusters predicted in strains LJC-15^T^, NGA-4^T^ and QZ2-6-4^T^ using antiSMASH.

## Abbreviations

The following abbreviations are used in this manuscript:

dDDH	digital DNA-DNA hybridization
ANI	average nucleotide identity
LB	Luria–Bertani
TSA	Tryptic soy agar
R2A	Reasoner’s 2A
NA	nutrient agar
ISP2	International Streptomyces Project 2
NJ	neighbor-joining
ML	maximum likelihood
DPG	diphosphatidylglycerol
PG	phosphatidylglycerol
BGCs	biosynthetic gene clusters
