# Supplementary material for: Agromyces capsici sp. nov., Agromyces cucurbitae sp. nov., and Agromyces solani sp. nov.: Three New Species Isolated from Rhizosphere Soils in Yunnan Province, China
Source: Microorganisms. 2026 Aug 12;14(8):1776. doi: 10.3390/microorganisms14081776 (PMC13515495; doi:10.3390/microorganisms14081776)
Supplement: Supplementary file 1 [file microorganisms-14-01776-s001.zip › Supplementary material-Agromyces.pdf]

Agromyces capsici sp. nov., *Agromyces cucurbitae* sp. nov., and *Agromyces solani* sp. nov.: three new species isolated from rhizosphere soils in Yunnan Province, China.

Zhongwen Duan[1,2,3], Shuailiang Shi[1,2,3], Bangli Huang[1,2,3], Mingji Yang[1,2,3], Yongxia Wang[4*], Zefen Yu[1,2*]

1 State Key Laboratory for Conservation and Utilization of Bio-Resources in Yunnan, Yunnan University, Kunming 650504, China

2 Yunnan Key Laboratory of Basic Research and Innovative Application for Green Biological Production, Yunnan University, Kunming 650504, China

3 School of Life Sciences, Yunnan University, Kunming 650504, China

4 Yunnan Institute of Microbiology, School of Life Sciences, Yunnan University, Kunming 650504, China

Corresponding authors: Yongxia Wang (wangyx@ynu.edu.cn); Zefen Yu (zfyu@ynu.edu.cn)

Figure S1. Polar lipid profiles of strains LJC-15^T, NGA-4^T and QZ2-6-4^T.
(a), using molybdophosphoric acid; (b), using molybdenum blue reagent; (c), using ninhydrin reagent; (d), using anisaldehyde reagent.
DPG, diphosphatidylglycerol; PG, phosphatidylglycerol; GL, unidentified glycolipids.

LJC-15^T

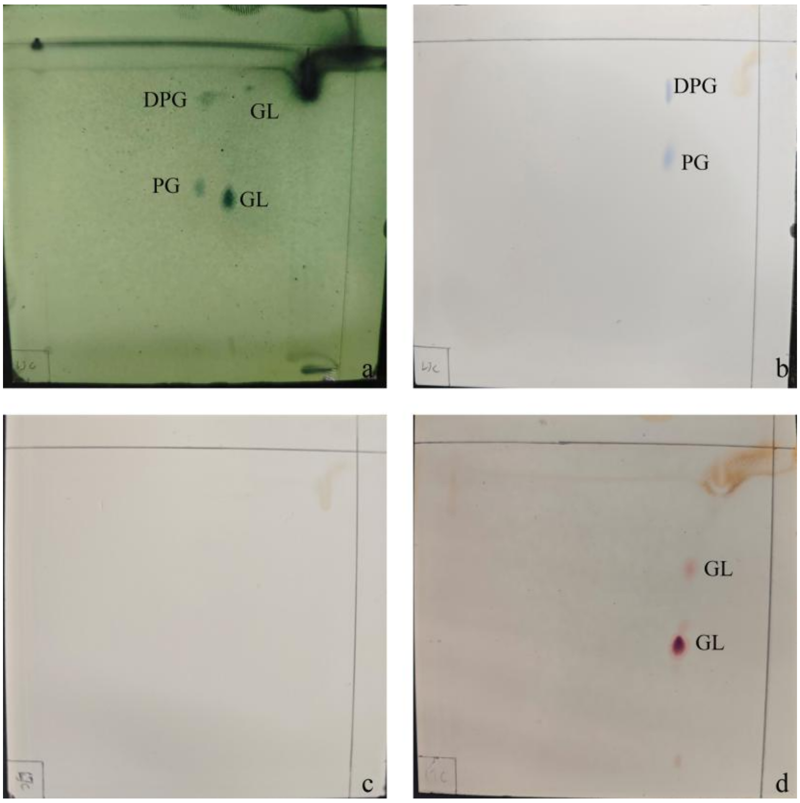

NGA-4^{T}

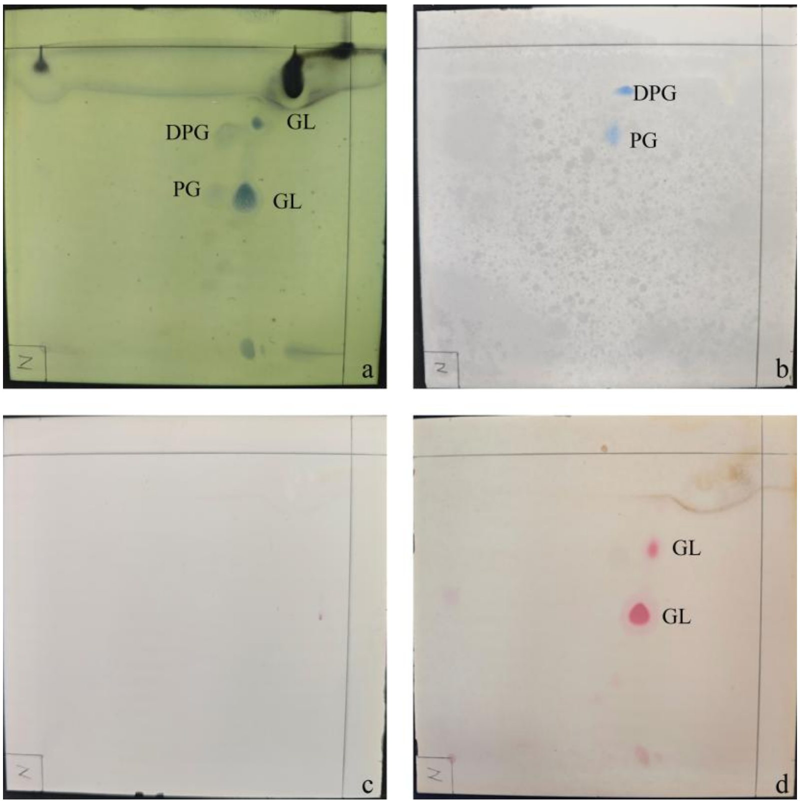

QZ2-6-4^{T}

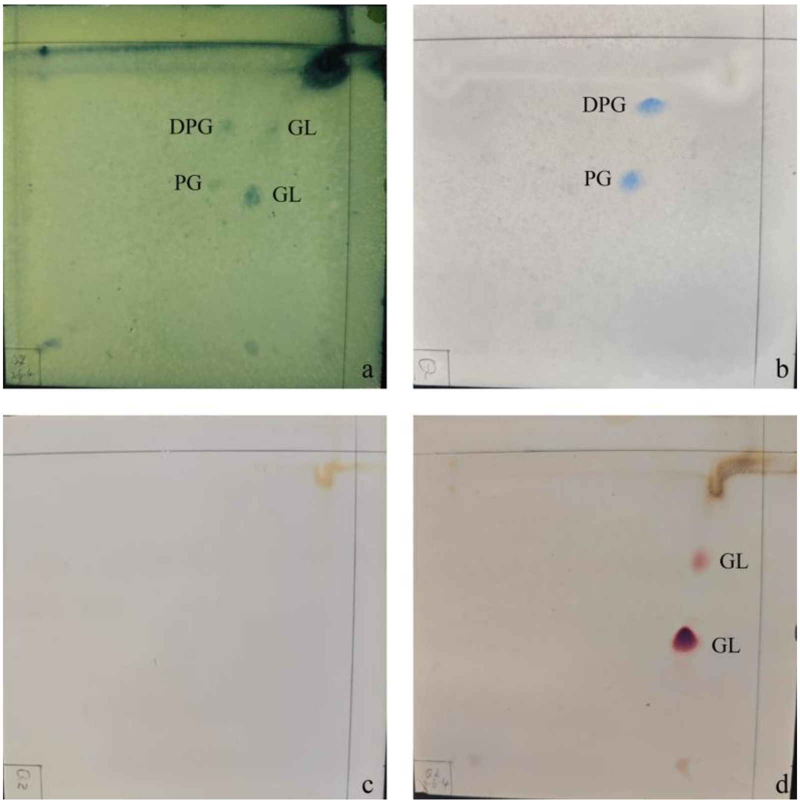

Figure S2. Maximum-likelihood (ML) phylogeny inferred from 16S rRNA gene sequences of the three novel strains (LJC-15^T, NGA-4^T, and QZ2-6-4^T) and selected type strains of the genus *Agromyces*. *Microbacterium lacticum* DSM 20427^T was used as the outgroup. Bootstrap percentages (1,000 replicates) are shown at nodes for values ≥50%. The scale bar indicates 0.01 substitutions per nucleotide position.

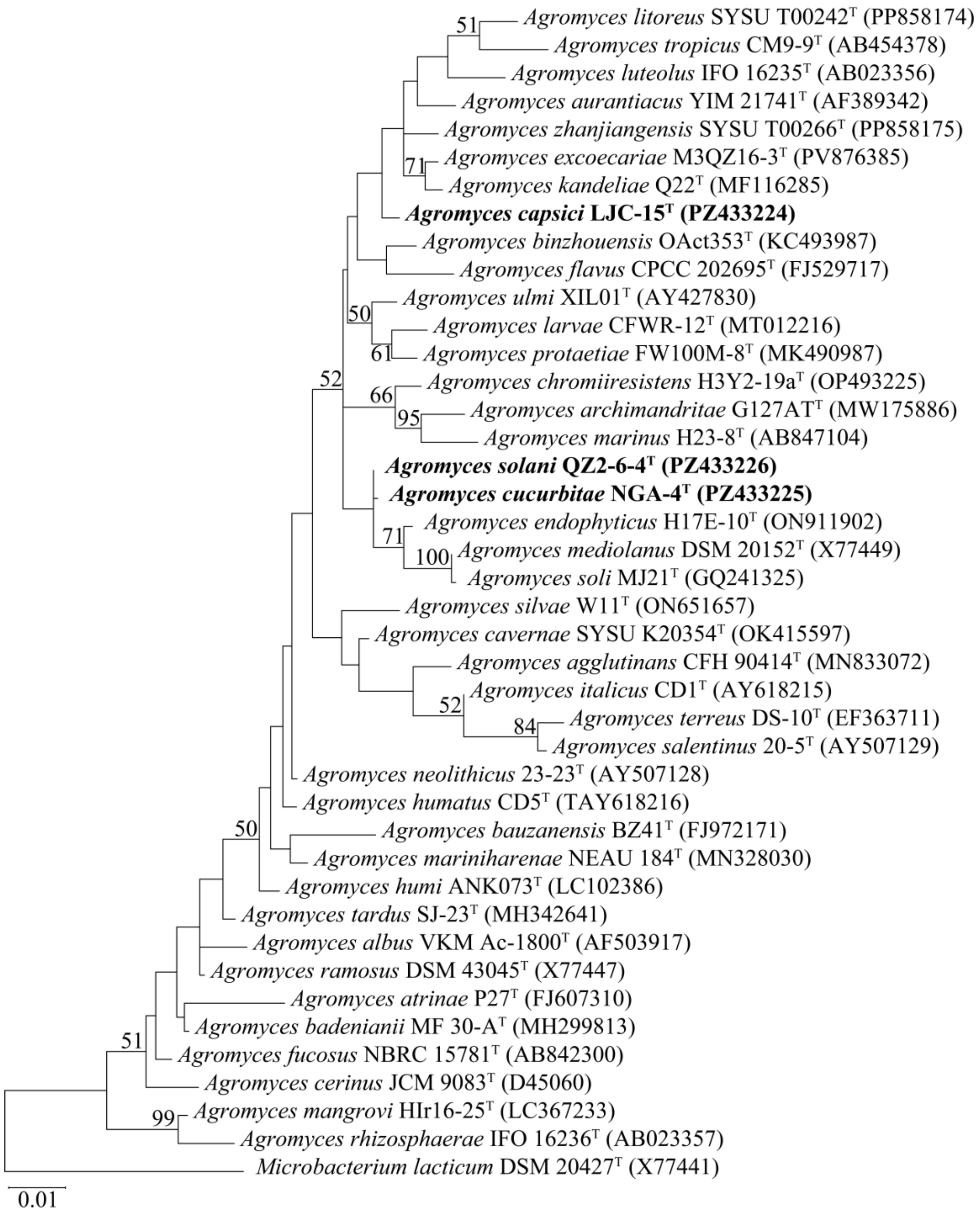

Table S1. Biochemical and enzymatic characteristics of strains LJC-15^T, NGA-4^T and QZ2-6-4^T determined using the API 20NE and API ZYM systems. +, positive; −, negative; W, weakly positive.

**Characteristics**		LJC-15^T	NGA-4^T	QZ2-6-4^T
**API 20NE**				
**NO3**	Reduction of nitrate to nitrite	-	+	-
**TRP**	Indole production	-	-	-
**GLU**	Fermentation of D-glucose	-	-	-
**ADH**	Arginine dihydrolase	-	-	-
**URE**	Urease	-	-	-
**ESC**	Aesculin hydrolysis	+	+	+
**GEL**	Gelatin hydrolysis	+	+	+
**PNPG**	β-galactosidase (PNPG)	+	+	+
**Assimilation of:**				
**GLU**	D-glucose	+	+	+
**ARA**	L-arabinose	+	+	+
**MNE**	D-mannose	+	+	+
**MAN**	D-mannitol	-	-	-
**NAG**	N-acetyl-D-glucosamine	+	+	+
**MAL**	D-maltose	+	+	+
**GNT**	Potassium gluconate	+	+	+
**CAP**	Capric acid	-	-	-
**ADI**	Adipic acid	-	-	-
**MLT**	Malic acid	-	-	-
**CIT**	Citrate	+	-	+
**PAC**	Phenylacetic acid	+	-	+
**API ZYM**				
2	Alkaline phosphatase	-	W	W
3	Esterase (C4)	+	+	+
4	Esterase lipase (C8)	+	+	+
5	Lipase (C14)	-	-	-
6	Leucine arylamidase	+	+	+
7	Valine arylamidase	+	+	+
8	Cystine arylamidase	+	+	+
9	Trypsin	-	-	-
10	α-Chymotrypsin	-	-	-
11	Acid phosphatase	W	W	W
12	Naphthol-AS-BI-phosphohydrolase	+	+	+
13	α-galactosidase	-	+	W
14	β-galactosidase	+	+	+

15	β-glucuronidase	-	-	-
16	α-glucosidase	+	+	+
17	β-glucosidase	+	+	+
18	N-acetyl-β-glucosaminidase	-	-	-
19	α-mannosidase	W	-	W
20	α-fucosidase	-	-	-

Table S2. A putative prophage region predicted in the genome of strain NGA-4^T using PHASTEST.

Strain	Region	Length	Completeness (score)	Region Position	Total proteins	Most common phage match	GC content (%)
**NGA-4^T**	1	35.2 kb	Intact (140)	552,670–587,917	51	PHAGE_Arthro_Eileen_NC_048094 (4)	69.01

Table S3. Selected phage-associated genes predicted within the putative intact prophage region of strain NGA-4^T.

CDS Position	Protein ID	Putative function	Top BLAST match (phage)	E-value
552773–553882	PP_00520	tyrosine integrase	Arthro_Mufasa8_NC_049478	3.21e-80
554063–554374	PP_00521	gp33	Mycoba_Che9c_NC_004683	1.68e-11
554446–554940	PP_00522	gp32	Mycoba_Che9c_NC_004683	2.08e-50
556865–557878	PP_00529	lysin A	Coryne_Poushou_NC_042139	5.85e-06
560851–561270	PP_00532	hypothetical protein	Caulob_CcrColossus_NC_019406	3.31e-05
561273–562169	PP_00533	hypothetical protein	Strept_phiCAM_NC_041856	7.7e-53
562178–562990	PP_00534	tail protein	Arthro_KellEzio_NC_031231	4.09e-13
562930–565107	PP_00535	tape measure protein	Arthro_Preamble_NC_041944	1.58e-79
565120–565422	PP_00536	hypothetical protein	Propio_PHL041M10_NC_027391	2.67e-05
565844–566653	PP_00538	major tail protein	Gordon_BritBrat_NC_030942	7.67e-24
567424–567765	PP_00541	head-to-tail stopper	Arthro_Kuleana_NC_049473	1.82e-05
567762–568133	PP_00542	hypothetical protein	Mycoba_Mendokysei_NC_051726	5.43e-20
568344–569159	PP_00544	major capsid protein	Mycoba_Poenanya_NC_051699	5.19e-42
569180–569743	PP_00545	putative head assembly scaffold protein	Propio_Stormborn_NC_027622	7.08e-10
569833–570549	PP_00546	capsid maturation protease	Gordon_Lucky10_NC_031267	4.74e-29
570533–571966	PP_00547	portal protein	Mycoba_Gaia_NC_026590	3.66e-101
571963–573369	PP_00548	terminase	Propio_PHL025M00_NC_027357	2.3e-57
574614–575672	PP_00551	helix-turn-helix DNA binding protein	Arthro_Constance_NC_048092	1.11e-26
575669–576370	PP_00552	hypothetical protein	Mycoba_Yoshi_NC_042030	3.33e-08
576881–577231	PP_00554	hypothetical protein	Arthro_Eileen_NC_048094	7.51e-06
577533–580013	PP_00555	DNA primase/polymerase	Arthro_Eileen_NC_048094	0.0
580099–580710	PP_00556	hypothetical protein	Mycoba_Amelie_NC_051613	5.56e-22

580725–581666	PP_00557	exonuclease	Arthro_Eileen_NC_048094	2.67e-64
581663–582286	PP_00558	phosphoesterase	Microb_Ilzat_NC_042111	3.77e-101
583202–584665	PP_00561	hypothetical protein	Microc_MaMV_DC_NC_029002	1.4e-05
585045–585404	PP_00563	DNA binding protein	Mycoba_Pipsqueaks_NC_042055	3.62e-37
586596–586856	PP_00568	excisionase	Gordon_Guacamole_NC_031265	9.46e-09
587414–587815	PP_00570	peptidase	Rhodoc_Sleepyhead_NC_048782	1.52e-12

Table S4. Resistance- and antibiotic-target-associated model matches identified in the genomes of strains LJC-15^T, NGA-4^T and QZ2-6-4^T using ARTS. Values indicate the numbers of model matches identified in each genome.

ARTS model ID	Model description	LJC-15^T	NGA-4^T	QZ2-6-4^T
RF0007	ABC_efflux	3	3	4
PF00044.19	Gp_dh_N	3	3	3
PF00185.19	OTCace	2	2	2
PF00204.20	DNA_gyraseB	2	2	2
PF00364.17	Biotin_lipoyl	9	7	7
PF00521.15	DNA_topoisoIV	2	2	2
PF01039.17	Carboxyl_trans	2	2	2
TIGR02013	TIGR02013	1	1	1
TIGR00663	TIGR00663	1	1	1
RF0050	Chlor_Acetyltrans_CAT	0	1	1
RF0155	vanS	0	1	1

Note: The presence of these model matches alone does not demonstrate an antimicrobial-resistance phenotype.

Table S5. Secondary metabolite biosynthetic gene clusters predicted in strains LJC-15^T, NGA-4^T and QZ2-6-4^T using antiSMASH.

Strain	Region	Type	Start (bp)	End (bp)	Most similar gene cluster	Similarity confidence
LJC-15^T	Region 1.1	T3PKS	900,849	941,997	Bisenarsan	Low
	Region 1.2	NAPAA	1,058,404	1,092,342	ε-poly-L-lysine	High
	Region 1.3	terpene-precursor	1,589,811	1,610,932	-	-
	Region 1.4	terpene-precursor	1,795,489	1,816,667	-	-
	Region 1.5	RRE-containing	2,404,665	2,424,940	-	-
	Region 1.6	NRPS-like	2,498,010	2,540,688	-	-
	Region 2.1	terpene; β-lactone	984,473	1,028,886	carotenoid	Low
NGA-4^T	Region 1.1	T3PKS	1	31,093	-	-
	Region 1.2	terpene	374,483	398,690	carotenoid	Low
	Region 1.3	NRPS-like	1,387,354	1,429,882	-	-
	Region 3.1	hydrogen-cyanide	77,942	90,714	-	-
	Region 3.2	terpene-precursor; β-lactone	628,962	664,579	-	-
QZ2-6-4^T	Region 1.1	hydrogen-cyanide	1,369,119	1,381,903	-	-
	Region 2.1	butyrolactone	82,265	93,461	-	-
	Region 2.2	NRPS-like	312,793	355,354	-	-
	Region 3.1	terpene	516,764	540,944	carotenoid	Low
	Region 3.2	T3PKS	878,670	908,892	-	-
	Region 4.1	β-lactone; terpene-precursor	45,761	81,346	-	-
